# Erythrocyte Stiffness during Morphological Remodeling Induced by Carbon Ion Radiation

**DOI:** 10.1371/journal.pone.0112624

**Published:** 2014-11-17

**Authors:** Baoping Zhang, Bin Liu, Hong Zhang, Jizeng Wang

**Affiliations:** 1 School of Civil Engineering and Mechanics, Lanzhou University, Lanzhou, 730000, PR China; 2 Key Laboratory of Mechanics on Disaster and Environment in Western China, The Ministry of Education of China, Lanzhou University, 730000, PR China; 3 Institute of Biomechanics and Medical Engineering, Lanzhou University, Lanzhou, 730000, PR China; 4 Department of Heavy Ion Radiation Medicine, Institute of Modern Physics, Chinese Academy of Sciences, Lanzhou 730000, PR China; University of California San Diego, United States of America

## Abstract

The adverse effect induced by carbon ion radiation (CIR) is still an unavoidable hazard to the treatment object. Thus, evaluation of its adverse effects on the body is a critical problem with respect to radiation therapy. We aimed to investigate the change between the configuration and mechanical properties of erythrocytes induced by radiation and found differences in both the configuration and the mechanical properties with involving in morphological remodeling process. Syrian hamsters were subjected to whole-body irradiation with carbon ion beams (1, 2, 4, and 6 Gy) or X-rays (2, 4, 6, and 12 Gy) for 3, 14 and 28 days. Erythrocytes in peripheral blood and bone marrow were collected for cytomorphological analysis. The mechanical properties of the erythrocytes were determined using atomic force microscopy, and the expression of the cytoskeletal protein spectrin-α1 was analyzed via western blotting. The results showed that dynamic changes were evident in erythrocytes exposed to different doses of carbon ion beams compared with X-rays and the control (0 Gy). The magnitude of impairment of the cell number and cellular morphology manifested the subtle variation according to the irradiation dose. In particular, the differences in the size, shape and mechanical properties of the erythrocytes were well exhibited. Furthermore, immunoblot data showed that the expression of the cytoskeletal protein spectrin-α1 was changed after irradiation, and there was a common pattern among its substantive characteristics in the irradiated group. Based on these findings, the present study concluded that CIR could induce a change in mechanical properties during morphological remodeling of erythrocytes. According to the unique characteristics of the biomechanical categories, we deduce that changes in cytomorphology and mechanical properties can be measured to evaluate the adverse effects generated by tumor radiotherapy. Additionally, for the first time, the current study provides a new strategy for enhancing the assessment of the curative effects and safety of clinical radiotherapy, as well as reducing adverse effects.

## Introduction

Ionizing radiation and radiotherapy commonly encounter problems not only due to the action of the treated tumor but also due to the adverse effects of irradiation on non-target cells or tissues [1]. Over the past decades, due to their high targeting ability and distinct curative effects, radioactive rays and ion beams have been intensively researched. Thus, special charged ion beam-carbon ion beams (CIBs) have great potential for the development of radiation therapy. Because CIBs have a special energy beam path, the so-called Bragg peak characteristic of its occurrence provides a higher relative biological effectiveness (RBE) than low linear energy transfer (LET) radiation (X-rays, γ-rays and electron beams) [2], [3]. Thus, heavy ion beams (^12^C^6+^ ions) can precisely target the nidus and avoid energy loss, leading to improved targeting with greater curative effect and better local control at the tumor site than is possible with conventional X-rays or similar treatments [4], [5]. However, although the beams have many advantages in their application, the adverse effects of treatment caused by the rays themselves remain an inevitable problem. Improving the maximum targeting of radiation therapy and reducing its adverse effects on normal cells remain major research focuses for tumor radiotherapy. Previous studies mainly emphasized the direct effects of cellular lethality caused by the irradiation of human organs or tissues [6] or investigated these aspects within the context of chromosomal rearrangement and mutation in the genetic material arising from energy deposition [7]–[9]. Additionally, whether the therapeutic success that is possible with X-ray treatment can also be achieved with heavy ion radiation is a major question.

The organs of the blood and hematopoietic systems are vulnerable to radiation [10]–[14]. Indeed, many studies have shown that radiation can cause a decrease in the visible components of blood [15], cell morphology changes, alterations to the electrolyte state in peripheral blood (PB), mild hyperplasia in the bone marrow (BM) [16], serious inhibition or even destruction of the bone marrow [17], [18], a reduction in the production of erythrocytes [19], changes in the aggregation state of platelets [20], and induction of the transformation of normal stem cells into tumor stem cells [20]–[22]. Thus, a hemogram of the blood system is often used initially as an approximate guide to assess the adverse effects caused by radiotherapy to the human body. Indeed, CIR may cause changes of in the hemogram and myelogram. Likewise, biological function and morphology must be in conformity with each other to determine the occurrence of disease. However, several investigations have indicated that radiation induces a series of complex and dynamic changes in cell shape as well as membrane damage and that a close relationship exists among the membrane skeleton components [23] and the function and antioxidant effects of electrolytes [9], [24]–such as changes in cell membrane permeability and alterations of the membrane and cytoskeleton–which can cause changes in the membrane, including organizational and metabolic changes [25], eventually leading to cell dysfunction.

Some considerable insights have already been obtained concerning the physiological or pathological processes of cell movement, invasion and migration through cell biomechanics research. Presently, cell mechanics are favored for understanding the problem. Additionally, not only is the cell membrane considered a very important factor in the response to damage; the cytoskeleton also plays a key role, being the most direct embodiment of cell morphology changes. For instance, the role of cytoskeletal morphological remodeling in tumor development is a crucial problem. Thus far, very few studies have assessed the biomechanical principles underlying the cytoskeletal morphological remodeling induced by radiation to evaluate the safety of ion radiotherapy in the human body through using morphological remodeling mechanics at the cellular level to understand how heavy ion radiation affects organs and tissues of the body from the perspective of biomechanics. This study investigates the mechanical properties of the membrane surface, cytoskeletal morphological remodeling and cytoskeletal protein levels to clarify the morphological remodeling process of the cytoskeleton. Normal erythrocytes have good mechanical properties that depend on an equilibrium state between two parameters–morphological motion and deformations–in particular, the geometric shape and biophysical characteristics of the spherical surface area, the viscosity of the cytoplasm, and the elasticity of the membrane. Alteration of any of these factors can lead to the differences in the cell membrane and the cytoskeletal structure. Previous studies have shown that some erythrocyte-related diseases are associated with the following three significant characteristics: the typical biconcave disc shape, the phospholipid bilayer membrane and the cytoskeleton meshwork. If these shape-changing agents cause area differences between the change in membrane morphology and mechanical properties of the cell, the process of the disease is altered [26]. Therefore, the size and shape, the phospholipid bilayer membrane and the cytoskeletal network govern the deformation and damage of erythrocytes and are widely considered to represent changes in biological behavior or function [27]–[29]. Typical illnesses related to erythrocyte dysfunction include malaria [30]–[34], sickle cell anemia [35], [36], hemolytic anemia [37], [38], cancer [39], spherocytosis and elliptocytosis [30], [40], and diabetes mellitus [41]. Thus, it has been vital to study the pathophysiology of these diseases [27], [42], [43].

Biological-type atomic force microscopy (BT-AFM) can allow the direct visualization of cell morphology changes in erythrocytes at the micro-nano level using an inverted microscope and measurement of the mechanical properties of biological specimens [44], [45]. However, measurement of the dynamic properties of cells as a characteristic feature can provide important information regarding their physical state [46], [47]. Current literature has suggested that altering the physical properties of the cytoplasm and membrane-cytoskeleton in erythrocytes can result in morphologically distinct signals [46], [48]. Thus, the remarkable changes in erythrocyte morphology result from a coupled dynamic response of the phospholipid bilayer membrane and an elastic spectrin molecular meshwork on the cytoplasmic face [27], [31], [42], [49]–[51]. Generally, erythrocyte damage depends on the phospholipid bilayer membrane of an adult red cell (mainly including membrane proteins or enzymatic erythrocyte defects), which is supported on its inner surface by a complex arrangement of spectrin-spectrin molecular interactions of cytoskeletal proteins (membrane skeleton). A major component of cytoskeletal proteins is the neofunctionalized protein spectrin, called an erythrocyte “ghost”, that was first reported to be isolated from human erythrocytes in 1968 by Marchesi and Steers [52], [53]. Studies have demonstrated that some examples of unhealthy cell morphology changes produce adverse outcomes, and these changes were focused on the interactions between the cell membrane and the cytoskeleton [50], [54], [55]. Additionally, previous theoretical research has contributed to a reliance on numerical modeling for the analysis of the membrane shape and surface free energy [48], [56]–[60], partly revealing the time-dependent relationship between the shape changes and deformability of erythrocytes [61], as well as displaying the elastic response of erythrocytes to small and large deformations [62]–[65]. Therefore, through quantitative measurement of the inherent behavior of erythrocytes, such as the elasticity of the cell membrane (cell stiffness), cell membrane contributions to the local or nonlocal bending deformation in the process of damage, changes in tension in the membrane-skeleton system, and the membrane surface area and size of erythrocytes [66], the degree of erythrocyte damage can be further assessed.

Herein, this paper will provide further insight into the adverse effects in erythrocytes related to radiation by examining the changes in microstructure and mechanical properties that have been used to analyze the functional relationship of erythrocytes before and after irradiation. We investigated whether subtle changes in the morphology and mechanical properties of cells can be used to assess the effects of radiation and to better depict the correlations among signal molecules involved in the configuration of the cytoskeleton, cell morphology changes, and the dynamic mechanical properties of cells in the process of damage. Based on these attempts, we focused on a new method of explaining the deformation or damage via the mechanical mechanism of erythrocyte morphological remodeling in different irradiated groups. Thus, according to the established parameters and these insights, we hypothesized the quantitation of the degree of erythrocyte damage to better explain cytoskeleton signaling dominated by biochemical molecules involved in the erythrocyte cytoskeleton morphological remodeling process, the biomechanical mechanism underlying erythrocyte induction by irradiation, and the relationship between the erythrocyte shape and injury rate to provide a theoretical base. In the present article, cell stiffness was chosen to meticulously estimate biological and adverse effects in erythrocytes during irradiation. Finally, this new perspective should be exploited to develop biomechanics to better assess the onset or progression of damage caused by ionizing radiation and to identify novel potential biomechanical targets or characteristics for therapeutic interventions.

## Materials and Methods

### Animals

SPF-class female Syrian golden hamsters (6–8 weeks old and weighing 80–100 g) provided by the Lanzhou Institute of Biological Products (Lanzhou, China) were used in the current study. All of the animals were maintained under standardized conditions (temperature, 22±2°C; humidity, 40±10%; light/dark cycle, 12 h:12 h) and given standard food and water ad libitum. The animal experimental protocols were approved by the Animal Experiment Medicine Center of Lanzhou University. A total of 156 hamsters were randomly divided into three groups: a carbon ion irradiation group (1 Gy, 2 Gy, 4 Gy, or 6 Gy; six hamsters per group), an X-ray irradiation group (2 Gy, 4 Gy, 6 Gy, or 12 Gy; six hamsters per group), and a control non-irradiated group (0 Gy; twelve hamsters per group).

### Irradiation procedure

Each hamster was positioned in a chamber attached to the irradiation equipment at the Heavy Ion Research Facility in Lanzhou (HIRFL, Institute of Modern Physics, Chinese Academy of Sciences, Lanzhou, China). The whole body of the hamster was irradiated with ^12^C^6+^ ion beams at an energy of 270 MeV/u and LET of 10 keV/m, with a dose rate of 0.3 Gy/min. When ^12^C^6+^ ion beams at 200 MeV/u and 31.3 keV/m of the beam entered the chamber, the ^12^C^6+^ ion dose of 0.5 Gy corresponded to a fluence of 3.0×10^7^ particles/cm^2^. The carbon ions were equipped with a passive beam delivery system. The data were controlled automatically by a microcomputer during irradiation. Particle fluences were determined from an air-ionization chamber signal according to the calibration of the detector (PTW-UNIDOS; PTW-Freiburg Co., Wiesbaden, Germany). Similarly, animals exposed to X-rays were given whole-body radiation using an X-ray therapy machine (Elekta BMEI Medical Equipment Co. Ltd, Beijing, China) at a source-to-surface distance (SSD) of 100 cm and a dose rate of 2 Gy/min [67].

### Preparation of erythrocytes in peripheral blood and bone marrow

The experiments complied with the laboratory animal disposal regulations of the Animal Ethics Committee at Lanzhou University. Animals were sacrificed at different time points to collect peripheral blood (PB) and bone marrow (BM) following treatment with different doses of radiation. Briefly, fresh peripheral blood and bone marrow were retrieved from the hamsters. First, the animals were anesthetized with 0.3% pentobarbital sodium at the injection concentration range of 30–35 mg/kg body weight. After adequate anesthetization, dissection was performed, the abdominal vein was exposed, and then the blood from the abdominal vein was retrieved using an anti-coagulated tube containing EDTA. After the plasma and buffy coat were removed, the erythrocytes were washed three times in an iso-osmotic HEPES-Ringer’s buffer [68] (centrifugation at 1000×*g* for 10 min at room temperature (RT)). Following the final wash, the erythrocytes were resuspended in HEPES-Ringer’s buffer (100 mM NaCl, 10 mM HEPES, 4 mM KCl, 1 mM CaCl_2_, 0.7 mM NaH_2_PO_4_, 0.6 mM MgSO_4,_ and 5 mM glucose) with 1 mg/mL of bovine serum albumin (BSA). The final dilution of the erythrocyte pellet ranged from 1∶10,000 to 1∶50,000, which was sufficient to separate individual erythrocytes by several cell diameters on a microscope slide. Erythrocyte ghosts were prepared from these samples by hemolysis using a standard procedure [69] and phosphate-buffered saline (PBS, 151.2 mmol/L NaCl, 5.6 mmol/L KCl, 5.48 mmol/L Na_2_HPO_4_, 0.32 mmol/L NaH_2_PO_4_, pH 7.4, osmotic pressure 300 mosmol/kg). First, one drop of the suspension was placed on a glass substrate treated with poly-L-lysine (P4832; Sigma) and was then incubated for 1 hour at 37°C. Next, one drop of 0.5% glutaraldehyde was added for 1.5 min to fix the erythrocyte membrane to the substrate. Finally, the pretreated cells were washed with phosphate buffer solution to remove the unbound cells and glutaraldehyde. The samples were immediately subjected to AFM.

The whole femurs of hamsters were dissected, and then the bone was cut from the knee to the hip buttock to better expose the bone marrow (BM). With a pointed tip, the area around the bone marrow was mixed evenly. The bone marrow was removed (approximately 0.5 mm^3^) with the tips of a pair of tweezers, and its color and status were then recorded to prior loading into 1.5–2.0 mL of serum in vitro. The bone marrow-serum mixture was mixed by aspiration with a glass dropper tip approximately 20 times until the mixture was evenly dispersed, carefully avoiding the production of bubbles. Finally, using a trace sample gun, 50 µl of the bone marrow fluid (depending on the amount of bone marrow retrieved) was removed to be applied to a centrifugal smear machine small funnel at 600–800 r/min using a 10-min medium speed centrifuge. The pre-coated glass slide with the cells was removed, and the follow-up test was completed.

### Cytological analysis of the proportion of irradiated erythrocytes in the bone marrow

BM smear samples were prepared on different of substrates using three glass slides: one slide was used for Swiss-Giemsa staining, another was used to determine the number of nucleated cells in the BM, and the third was counted at low magnification. The BM cytological report contained four grades of activity: hyperactive, active, reduced and significantly reduced. Additionally, the erythroid cells in the BM smear were classified into four clinical types–pronormoblasts, basophilic erythroblasts, polychromatophilic erythroblasts and normoblasts–and then the proportion of the total number of nucleated cells was determined under oil. According to cell counting principles, the samples were divided into three types–low, medium and high cell count. Additionally, each slide was observed no fewer than 30 times, and each observation involved the selection of several fields for statistical processing parameters.

### Atomic force microscopy analysis of the changes in morphology and mechanical properties in erythrocytes induced by CIR or X-rays

A NanoWizard III AFM (JPK Instruments, Germany) mounted on an inverted optical microscope (Axiovert 200; Carl Zeiss Microimaging) was used for cell imaging and force spectroscopy. Silicon nitride cantilevers (PNP-DB; NanoWorld) with a nominal spring constant of 0.06 N/m (f_o_: 17 kHz) were used in all of the experiments. For each cantilever, the spring constant was determined using the thermal noise constant calibration method. To ensure reproducibility in force application, the cantilever sensitivity and spring constant were calibrated before each experiment using JPK Instruments software 4.2.61. The optical microscope was used to select the desired cell and position the AFM tip. All of the AFM images and measurements were obtained in contact mode in air.

### AFM imaging and measurement

**Figure 1:**
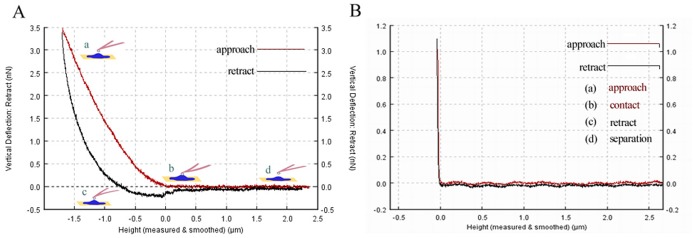
Force distance curve taken on the erythrocyte, Extend (or approach) (dark red) and retract (dark) curve clearly show hysteresis owing to the viscous and plastic behavior of the erythrocyte. (**A**) Cantilever deflection dependence on the tip sample distance (Z coordinate), and determination of the force-versus-indentation curves for control erythrocytes. (**B**) The straight line corresponds to curves measured on hard, non-deformable surface (glass coverslip) as the calibration curves. Lowercase letters show the marching trajectory of the probe in the whole process, and it consists of four steps, approach, contact, retract and separation (in **A**).

Erythrocyte imaging was performed on glass slides at RT. The AFM had a maximum scanning range from 50×50 µm bar (Fast × Slow) to 5×5 µm bar (Fast × Slow) and a vertical range (Z-direction) of 15 µm. Pretreated cells were imaged using a pixel resolution of 512 pixels at a line rate of 0.10–0.30 Hz. The imaging parameters were carefully monitored to achieve good contrast with scanning forces as determined from force distance curves in the experiment. Elastic modulus (cell stiffness) determination is detailed in Section 2.5.2 below. Regarding radiation dose changes, a series of 50–100 curves of elastic modulus measurements in the erythrocytes was performed for each specimen in which the probe was placed over the sample surface and then pushed against the sample. The force acting between the tip and the sample caused a deflection of the cantilever that was recorded as a function of the relative sample position as a force curve. **Figure 1** shows two curves, one obtained from a soft erythrocyte and the other from a hard material. After subtraction of these two curves, the force-versus-indentation curve was obtained. In this experiment, the glass coverslip was taken as a zero-reference hard surface and was used for calibration, and the calibration curve was recorded each time together with a newly prepared erythrocyte sample. Ultimately, all of the AFM data were acquired and analyzed using two proprietary codes–a topographical image analysis code and a code for force curve analysis–using JPK instruments data processing software version 4.2.61.

### Elastic modulus determination

Using AFM for nanoindentation is a useful tool to determine elastic properties such as the elastic modulus of biological samples [61], [70]–[73]. Cantilevers serve as soft nanoindenters that allow local testing of small and inhomogeneous samples such as cells or tissues. To calculate the parameter of interest, various models are used, but most of them are based on the Hertz model, modified to match the experimental conditions concerning the indenter’s shape or the thickness of the sample [74]–[76] **.** The cell elastic modulus was calculated by applying the modified Hertz model contact theory to the force curves [35], [75], [77], [78]. Thus, it is assumed that the surface is continuous, frictionless and incompressible (similar to rubber) at small deformations [79], and also consider the indentation is considered to be negligible compared with the sample thickness, to reflect the real situation of the cells and to ensure that the indentation depth is optimized. The Hertz model is valid for small indentations (5–10% of the height of the cell, approximately, 200–500 nm) where the substrate does not influence the calculations [45], [78], [80]. There may be additional limitations in the indentation depth if the tip shape model is an approximation.

In the present study, tiny force probes were employed during the entire experimental process, and the probes were modified by attaching 6-µm diameter polystyrene microspheres (Base Line Chromtech Research Centre, Tianjin, China) onto the cantilever with epoxy resin glue (Epoxy F-05 Clear; Alteco, Japan). Additionally, the cell deformation nonlinearity was reduced due to a more homogeneous contact between the cells and probe. Elastic modulus measurements were derived from the force-distance profiles (extended curves) acquired at different locations on the cells, and JPK instruments software was used for this purpose. Microprobes were positioned on the cell membrane under optical control, in accordance with the method described by Ketene [81], and force curves were acquired at a sampling rate of 5 kHz and a constant approach velocity of ∼1.0 µm/s. The movement is sufficiently slow that the viscous contributions are small, and force measurements are dominated by elastic behavior [82], [83]. A maximum force of 3.5±1.0 nN was implemented for all of the force curves to maintain a basis for comparison among the cells. In light of these claims, the parabolic model is often used if the indenter is a sphere because it is relatively easy to fit and yields a reasonable approximation for small indentations. Because the tip that comes in contact with the cell sample is spherical in shape, the model [84] predicts the relationship between the applied force and the indentation depth of the AFM cantilever into the soft sample by the following equation:
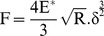
(1)where F is the applied force, R is the radius of the sphere, *δ* is the indentation, and E^*^is the relative Young’s modulus term:
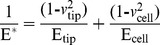
(2)Here, *v* is Poisson’s ratio, and *E*_tip_ and *E*_cell_ represent the Young’s modulus of the tip and the cell, respectively. If the assumption of an infinitely hard needlepoint tip is employed, then *E*_tip_>> *E*_cell_. Thus, equation (2) could be simplified to the following:
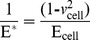
(3)where *ν* depends on the material. For soft biological samples (i.e., cells, soft tissue, lipid bilayers and vesicles) [35], Poisson’s ratio was assumed to be 0.5 in accordance with the incompressibility assumption usually employed for cells. Finally, the Hertz model equation assumes the following format:



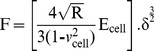
(4)However, regarding equation (4), it is difficult to determine the exact point at which the tip and the cell membrane come into contact, resulting in an inability to judge the most appropriate force curve to which the Hertz model is fitted. As a result, the following equation can be applied:
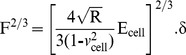
(5)


Additionally, when the dependence of the deformation on the force becomes linear, we use the indentation (*δ*) described as the difference in the relative changes of the piezo scanner displacement (*z*) and cantilever deflection (*d*). We obtain the following equation by replacing *δ*:
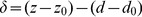
(6)where *z_0_* and *d_0_* are both the x and y coordinates of initial contact between the tip and the cell sample to identify the initial contact point. To attain the use of visual means to approximate the contact point for the fit region range, we adopt a semi-automated, mathematical approximation for the contact point. To better calculate the Young’s modulus, we substitute equation (6) into equation (5) to produce the formula below:



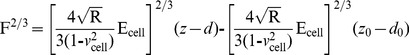
(7)Thus, this equation can be analyzed as a line in the (F^2/3^, z–d) plane, from which the Young’s modulus (E) can be directly calculated from the linear slope and contact point (z_0_-d_0_) through the intercept of Eq. (7) [82], [85]–[87]. In addition, the Hertz model assumes linearity, so it can be further analyzed according to the following formula:
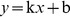
(8)


The slope information of the indentation curve (k) was used to calculate the Young’s modulus (E):
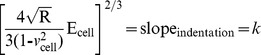
(9)


Solving for E_ cell_, we obtain the following:
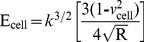
(10)


Equation (7) should give a good linear fit line for the indentation data. The Young’s modulus of the cell is considered a constant within the range of force applied to it. Presently, the contact point can be approximately represented by the b term in the following equation:
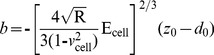
(11)


However, *z_0_* and *d_0_* are both the x and y coordinates of the initial point of contact. *d_0_* refers to the deflection offset that is experienced during force curve acquisition and can be determined conveniently by simply observing the raw deflection *vs.* piezo position data. We solve for *z_0_* as follows:
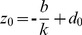
(12)


Ultimately, we will acquire a more accurate fitting region (*z_0_*, *d_0_*) for the Hertz model. All of the data analysis and curve fitting of the Hertz model to the collected force-indentation data were performed using JPK IP software, which offered automatic fitting for all of the indenter shapes. Finally, the elastic modulus (E) calculations were performed using the JPK IP software.

### Extraction and purification of scaffolding protein in irradiated erythrocytes, and western blot analysis of the expression of spectrin-α1

First, the erythrocytes were purified within 48 hours from hamster whole blood and were then isolated by centrifugation at 600×*g* for 20 min at 4°C. After removing the plasma and buffy coat by careful aspiration, the erythrocyte pellet was resuspended in cold isotonic buffer (145 mM NaCl, 5 mM KCl, 5 mM HEPES, pH 7.4) and then centrifuged again at 2,000×*g* for 10 min at 4°C. Thereafter, the erythrocyte suspension was washed with isotonic buffer four times to completely remove the plasma and buffy layers. Next, to remove white blood cells from the erythrocyte suspensions, the samples were passed through white blood cell filters (Leukotrap RC; Pall Corporation, East Hills, NY) twice. In each step of the purification process, the upper layer was removed. The erythrocytes were finally resuspended in cold isotonic buffer at pH 7.4, made up to a hematocrit of approximately 50%, and stored at 4°C until further analysis.

Erythrocyte samples were purified as described above and were incubated for 10 min at 4°C in buffer containing 1% Triton X-100, 100 mg/mL PMSF, and 1 mM EDTA. The cell lysates were then centrifuged at 30,000×*g* at 4°C. The obtained supernatants and pellets, as well as the control (intact cell suspension in PBS), were treated with sample buffer (125 mmol/L Tris-HCl, pH 6.8, 2% sodium dodecyl sulfate (SDS), 10% β-mercaptoethanol, 10% glycerol, and 0.01% bromophenol blue) and heated at 100°C for 8 min. Standard immunoblot procedures were used for all of the samples using the indicated antibody combinations. The protein samples were subjected to 6% SDS-PAGE gel electrophoresis followed by electrotransfer onto PVDF membranes in 0.2 M glycine-NaOH transfer buffer with 0.01% SDS. Filters were blocked for 2 h at RT with blocking buffer (10% FSC, 0.1% Tween-20, and PBS) and subsequently incubated for 2 h at RT in blocking buffer containing monoclonal spectrin-α1 primary antibody (sc-15371; Santa Cruz Biotechnology) at a dilution of 1∶1,000. Membranes were washed and incubated for 2 h at RT in blocking buffer containing 1∶10,000 affinity-purified goat anti-rabbit IgG (H&L) antibody (7074; Cell Signaling Technology) secondary antibody and then were extensively washed. Next, the antibody conjugate was decanted and washed for 30 min with agitation in wash buffer (TBS with 0.1% Tween-20), changing the wash buffer every 5 min. Protein visualization was performed using ECL reagents (Super Signal West Pico Chemiluminescent Substrate; 34077; Pierce) followed by photography. Normal untreated cells with extraction buffer were used as controls. Western blot analysis was performed using a color scanner by densitometry (HP Scan jet Enterprise 7000n) and analyzed using the Image J software (NIH).

### Statistical analysis

Except where noted, data are reported as the mean ± standard error, and statistical comparisons were performed using one-way ANOVA followed by the Tukey-Kramer HSD test for pair-wise comparisons. *p* values less than 0.05 were considered significant. All statistical analyses were performed using SPSS 11.5 (Statistical Product and Service Solutions) (Stanford University, USA).

## Results

### 3.1 Radiation-induced morphological changes of erythrocytes in hamster peripheral blood via optical microscope observation

**Figure 2:**
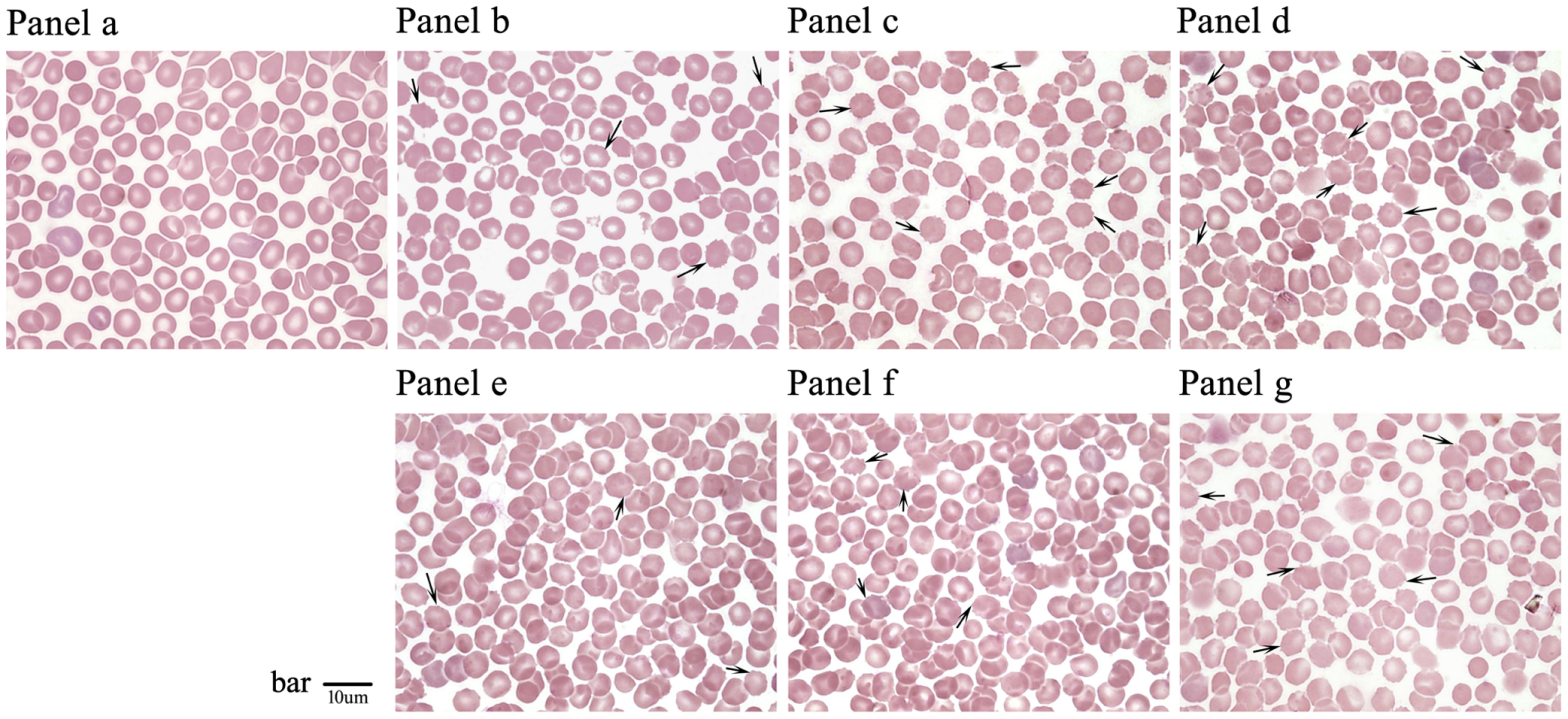
Photographs of analysis the changes of gross morphology of erythrocytes in blood smear at 3d after whole-body exposure to^ 12^C^6+^ ions or X-rays (by 100×oil immersion observation). Panel **a**: Non-irradiated control group erythrocytes (0 Gy); Panel **b**–**d**: erythrocytes of carbon ions radiation groups (Radiation dose: 2 Gy, 4 Gy and 6 Gy); and Panel **e**–**g**: erythrocytes of X-rays radiation groups (Radiation dose: 4 Gy, 6 Gy and 12 Gy). Black arrows indicate the morphological changes of erythrocytes. The scale bar is 10 µm.

Morphological changes were observed in normal and irradiated erythrocytes by optical microscopy. Non-irradiated erythrocytes in peripheral blood were typically biconcave discs, resembling round cakes that were thinner at the center and thicker at the edges. However, after irradiation with CIR for 3 days, these cells appeared to have undergone shrinkage and were irregular, and the edges of some cells exhibited abnormal spines or ruffles. As shown in **Figure 2**, these changes were associated with decreased erythrocyte levels, and the number of abnormally shaped cells increased gradually. Moreover, quantitative analysis was performed on the inherent physicochemical parameters of the erythrocytes, such as the size, shape, effective area of the membrane, and average volume of the cells after irradiation by CIR and X-rays. The results showed that these morphological changes were closely correlated with ionizing irradiation, and a dose-time effect relationship was observed in the irradiated group of erythrocytes with respect to the different radiation doses (**Table 1**).

**Table 1:** Measurement of the changes of erythrocytes shape by induced of ^12^C^6+^ ions and X-rays radiation in *Mesocricetus auratus.*

Group	Cells No.	Length(µm)	Width(µm)	Perimeter(µm)	Thickness(µm)	ROI area(µm^2^)	Volume(µm^3^)
**Control 0Gy**	1500	7.25±0.13	6.12±0.10	584.14±36.78	2.0	20517.44±3651.02	41.03±7.30
**^12^C^6+^ions 2Gy**	410	6.85±0.15^▴^	5.96±0.20	563.95±57.72^▴^	2.0	19019.15±3120.58	38.04±6.24^▴^
**4Gy**	376	6.72±0.16^▴^	5.85±0.11^▴^	547.98±47.86^▴^	2.0	18178.41±2933.45	36.36±5.87^▴^
**6Gy**	373	5.96±0.24^▴^	5.55±0.15^▴^	529.38±40.77^▴^	2.0	17317.20±2419.97	34.63±4.84^▴^
**X-rays 4Gy**	652	7.0±0.11^▴▴^	5.8±0.16^▴▴^	570.7±57.43^▴▴^	2.0	19496.54±2975.36	38.99±5.95^▴▴^
**6Gy**	325	6.8±0.15^▴▴^	5.6±0.12^▴▴^	535.6±37.50^▴▴^	2.0	18659.76±2260.22	37.32±4.52^▴▴^
**12Gy**	554	6.1±0.18^▴▴^	5.4±0.10^▴▴^	519.0±45.65^▴▴^	2.0	17734.23±2747.42	35.47±5.49^▴▴^

**Note**, Measurement of the morphological differences of erythrocytes had indicated the obvious impact of ^12^C^6+^ ions or X-rays radiation with Image J software. And these general varies mainly includes the physical and chemical parameters of the erythrocyte, such as length, width, perimeter, thickness, ROI area and volume (of average value). Because of the average thickness of erythrocytes in mammals is around in the range of 1.0–2.5 µm, and using an optical microscope in the experiment, so as to calculate the volume, we taken the average thickness value of 2.0 µm to obtain the relative size of erythrocytes. And in the list of **Tab**., ^▴^ Black triangle symbols significant statistical significance compared with the control group in carbon ion radiation (*p<0.05*), but ^▴▴^ double triangle line represent the differences results by inducing X-rays (*p<0.05*). (In detail, for length, Control *vs* C ions-2Gy, p = 0.000, *p<0.05;* Control *vs* C ions-4Gy, p = 0.000, *p<0.05;* Control *vs* C ions-6Gy, p = 0.000, *p<0.05;* Control *vs* X rays-4Gy, p = 0.000, *p<0.05;* Control *vs* X rays-6Gy, p = 0.000, *p<0.05;* Control *vs* X rays-12Gy, p = 0.000, *p<0.05;* for width, Control *vs* C ions-2Gy, p = 0.479, *p>0.05;* Control *vs* C ions-4Gy, p = 0.000, *p<0.05;* Control *vs* C ions-6Gy, p = 0.000, *p<0.05;* Control *vs* X rays-4Gy, p = 0.000, *p<0.05;* Control *vs* X rays-6Gy, p = 0.000, *p<0.05;* Control *vs* X rays-12Gy, p = 0.000, *p<0.05;* for perimeter, Control *vs* C ions-2Gy, p = 0.002, *p<0.05;* Control *vs* C ions-4Gy, p = 0.000, *p<0.05;* Control *vs* C ions-6Gy, p = 0.000, *p<0.05;* Control *vs* X rays-4Gy, p = 0.027, *p<0.05;* Control *vs* X rays-6Gy, p = 0.000, *p<0.05;* Control *vs* X rays-12Gy, p = 0.000, *p<0.05;* for volume, Control *vs* C ions-2Gy, p = 0.000, *p<0.05;* Control *vs* C ions-4Gy, p = 0.000, *p<0.05;* Control *vs* C ions-6Gy, p = 0.000, *p<0.05;* Control *vs* X rays-4Gy, p = 0.000, *p<0.05;* Control *vs* X rays-6Gy, p = 0.000, *p<0.05;* Control *vs* X rays-12Gy, p = 0.000, *p<0.05.*).

### 3.2 Influence of the proportion of erythroid cells in bone marrow exposed to radiation

**Figure 3:**
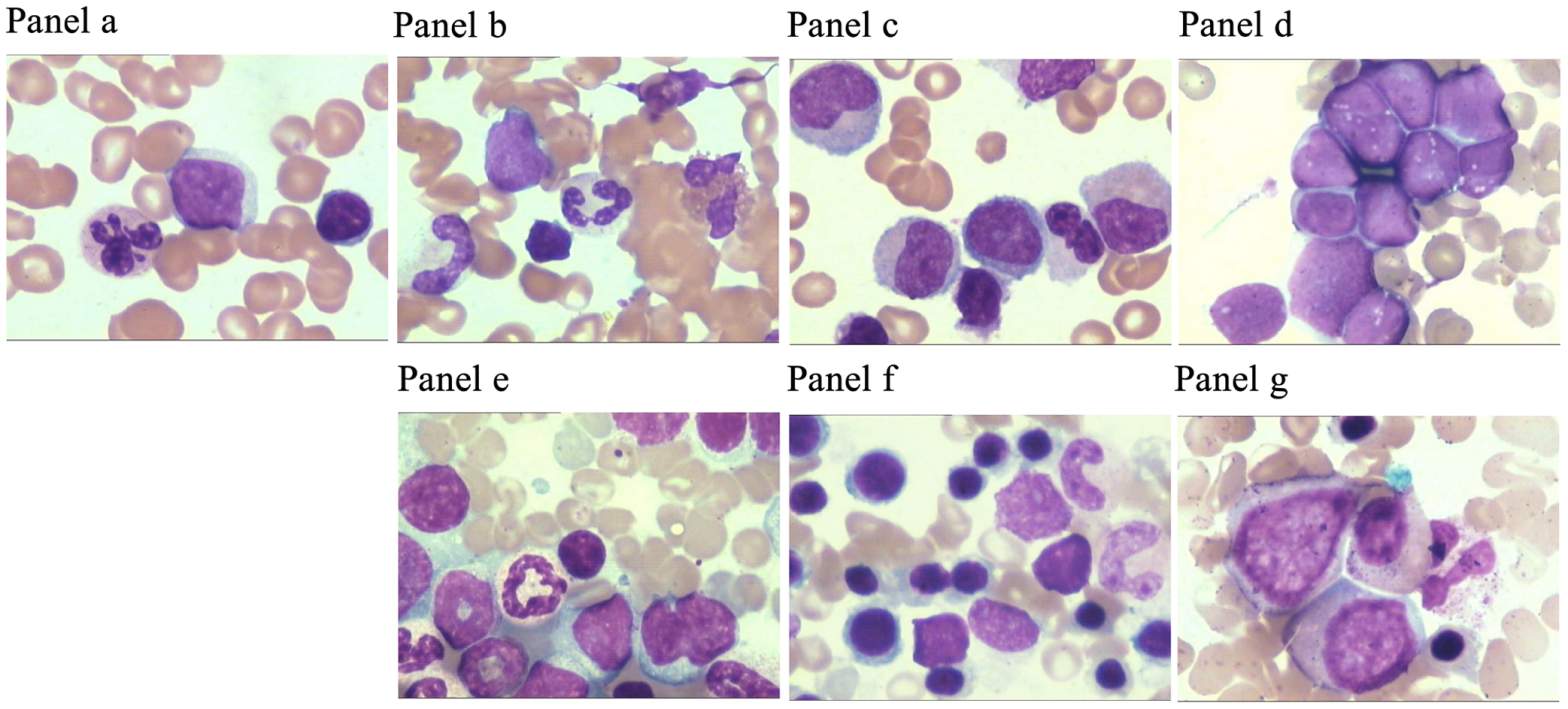
The distribution characteristics of erythrocytes in BM at 3d after exposed by different doses of ^12^C^6+^ ions or X-rays. Cytology analysis the distribution of erythrocytes in BM. In detailed, Panel **a**: Non-irradiated control group erythrocytes (0Gy); Panel **b**–**d**: erythrocytes of carbon ions radiation groups (Radiation dose: 2Gy, 4Gy and 6Gy); panel **e**–**g**: erythrocytes of X-rays radiation groups (Radiation dose: 4Gy, 6Gy and 12Gy). And BM aspirate smear from irradiated golden hamster showing abnormal erythroblasts indicative of a cell-division defect, or dysplasia. The cytology image was observed with 100×oil immersion, in the picture, pink represents the erythrocytes.

**Figure 4:**
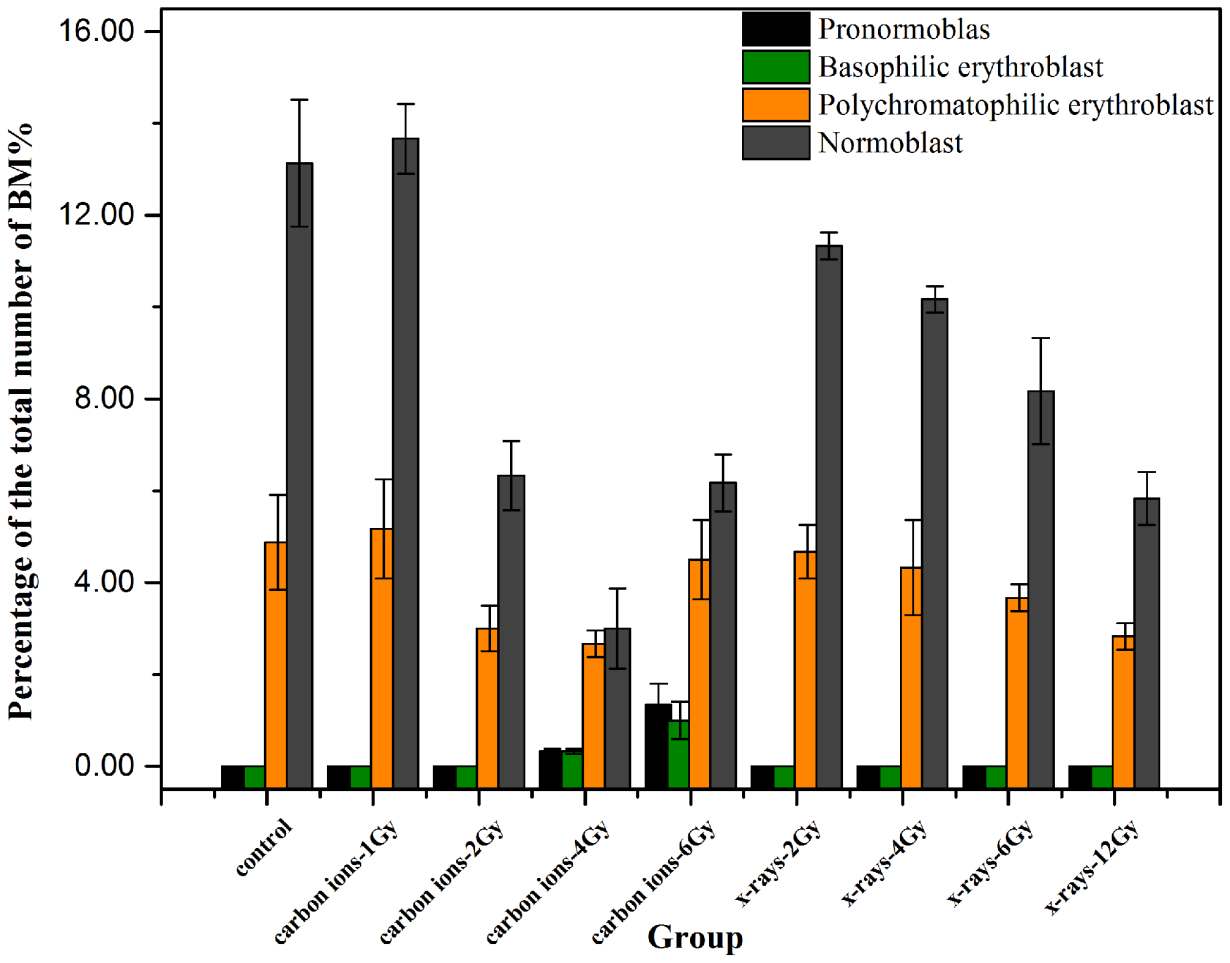
The number of erythroid cells in BM accounted for the relative percentage of the total of BM cells after exposed carbon ion beams or X-rays. From the column chart showed that the pronormoblast and basophilic erythroblast seemed upward tendency with the increasing of absorbed dose in carbon ions radiation, but the change in X-rays radiation groups seemed to be not obvious, as for the polychromatophilic erythroblast and normoblast, the percentage of cells number had decreased significantly in two types of irradiation, and the differences between groups had significant statistical significance (*p<0.05*).

Changes in the BM erythrocytes after irradiation were analyzed using a tangible cell-counting method. The purpose of our observations was to compare the characteristics among the different cell population types in the total number of BM cells and to understand the distribution characteristics of erythroid cells in BM caused by irradiation. The results revealed a mild myeloproliferative effect with small doses of radiation. Additionally, given the priority of the early development of erythroid cells, the pronormoblasts and basophilic erythroblasts seemed to demonstrate a tendency to increase with increasing absorbed doses of CIR; however, this change in the X-ray radiation group was not obvious. When the radiation dose increased, an obvious inhibitory effect was shown on BM. However, the proportions of polychromatophilic erythroblasts and normoblasts decreased significantly following both types of irradiation. In addition, the data indicated the presence of the myeloid maturation stage in a significantly higher proportion of granulocytes. Furthermore, the annular core, class changes and lack of particles were easily observed via the additive pleochroism of the erythrocytes (**Figure 3** and **Figure 4**)**.**

### 3.3 Alterations of erythrocyte morphology and mechanical properties triggered by radiation evaluated using atomic force microscopy

#### 3.3.1 AFM topographic imaging

**Figure 5:**
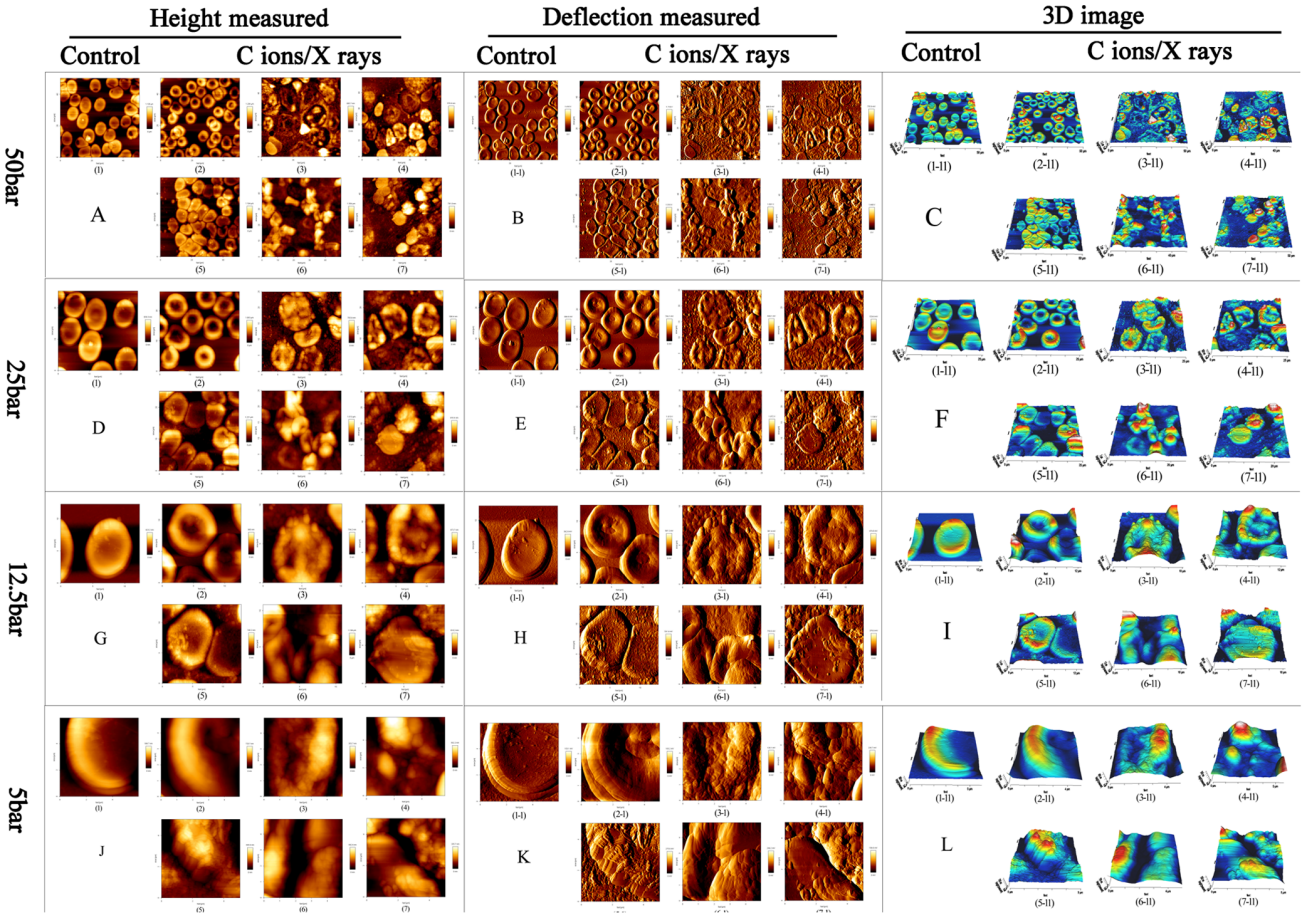
Effects of radiation on micromorphology in erythrocytes membrane. (**A–L**) Surface imaging of erythrocytes induced by carbon ion beams and X-rays radiation using biological type atomic force microscopy; and the graph **A**, **B** and **C** showed respectively for the Height measured image, Deflection image and 3D image at scan scale of 50 µm (Fast)×50 µm bar (Slow), [for **A**:(1) Control; (2) C ions 2Gy; (3) C ions 4Gy; (4) C ions 6Gy; (5) X-rays 4Gy; (6) X-rays 6Gy; (7) X-rays 12Gy; for **B**: (1–1) Control; (2–1) C ions 2Gy; (3–1) C ions 4Gy; (4–1) C ions 6Gy; (5–1) X-rays 4Gy; (6–1) X-rays 6Gy; (7–1) X-rays 12Gy; for **C**: (1–11) Control; (2–11) C ions 2Gy; (3–11) C ions 4Gy; (4–11) C ions 6Gy; (5–11) X-rays 4Gy; (6–11) X-rays 6Gy; (7–11) X-rays 12Gy]. But the graph **D**–**F** showed respectively for the Height measured image, Deflection image and 3D image at scan scale of 25 µm (Fast) ×25 µm bar (Slow); the graph **G**–**I** showed respectively for the Height measured image, Deflection image and 3D image at scan scale of 12.5 µm (Fast) ×12.5 µm bar (Slow); the graph **J**–**L** showed respectively for the Height measured image, Deflection image and 3D image at scan scale of 5 µm (Fast) × 5 µm bar (Slow). In addition, for a smaller scan scale was the same grouping as 50 µm (Fast) × 50 µm bar (Slow) bar. Here, the schematic showed different doses and type radiation for the influence of micromorphology of erythrocytes, and this fine change was captured by AFM.

AFM experiments were performed on erythrocytes that were spin-coated onto glass slides, representing the surface effects of the measurement period. Image acquisition was performed in contact mode, and the mechanical properties of the erythrocytes were measured using the force spectrum of the AFM. The AFM probe not only senses the differences in the surface and local mechanical properties in the cell, it also leads to differential surface deformation and contributes to image contrast. During AFM raster scanning, the AFM tip presses against the soft cell membrane into the cytosol until the membrane is resisted by the underlying stiffer structures, resulting in the sub-membrane structures appearing elevated and being detected in deflection images. Thus, through the analysis of AFM topographic images, including height-measured images, deflection images and 3D images, the imaging results can connect the membranous structure and function to reveal the variations in the microstructure of erythrocytes exposed to radiation. Unaffected erythrocytes are typically smooth, biconcave discs; when the cells were irradiated, a series of changes, ranging from minute to major, was captured throughout the whole process using BT-AFM, as detailed below (**Figure 5**).

#### 3.3.2 AFM measurement of the elastic modulus (cell stiffness) of irradiated erythrocytes

**Figure 6:**
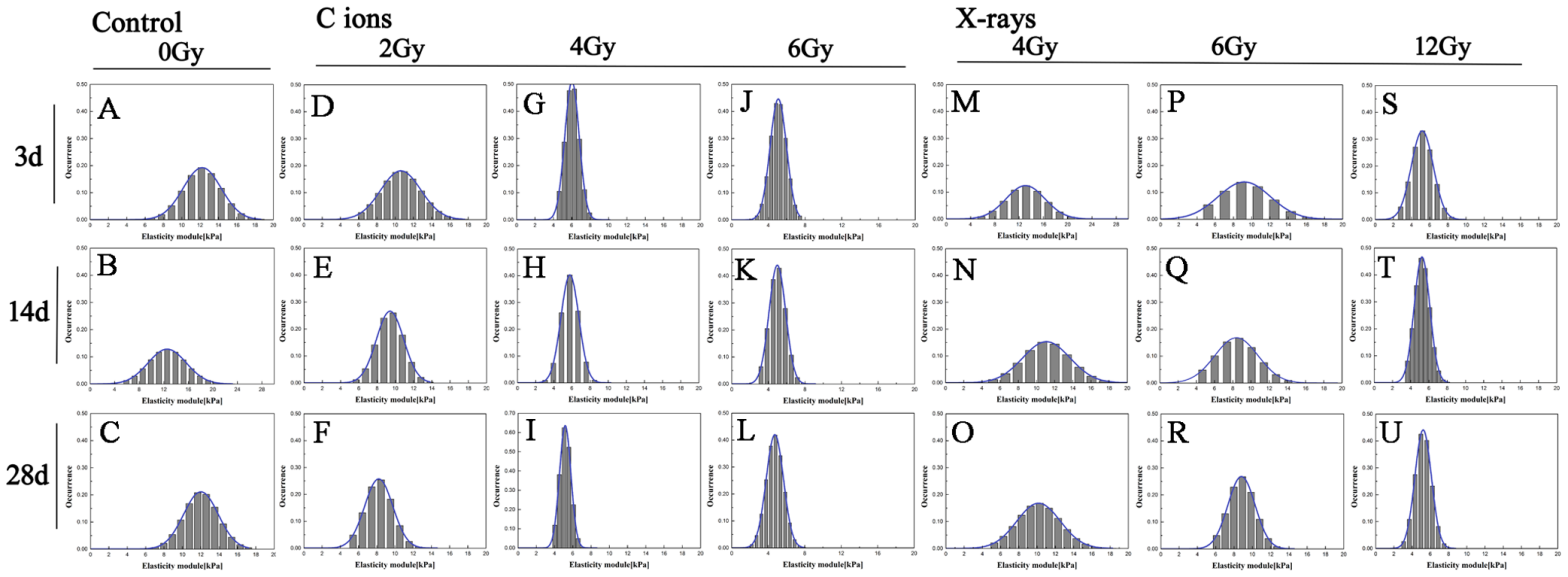
Histograms of erythrocytes elasticity modulus distributions induced by C ions or X-rays exposed for 3, 14 and 28 days. The bar graph (**A**), (**B**) and (**C**) represented control erythrocytes (0 Gy), 3d, 14d and 28d, respectively; the bar graph (**D**), (**E**) and (**F**) represented carbon ions 2 Gy, 3d, 14d and 28d, respectively; the bar graph (**G**), (**H**) and (**I**) represented carbon ions 4 Gy, 3d, 14d and 28d, respectively; the bar graph (**J**), (**K**) and (**L**) represented carbon ions 6 Gy, 3d, 14d and 28d, respectively. But the bar graph (**M**), (**N**) and (**O**) showed X-rays 4 Gy, 3d, 14d and 28d, respectively; the bar graph **(P**), (**Q**) and (**R**) showed X-rays 6 Gy, 3d, 14d and 28d; the bar graph (**S**), (**T**) and (**U**) showed X-rays 12 Gy, 3d, 14d and 28d, respectively.

**Figure 7:**
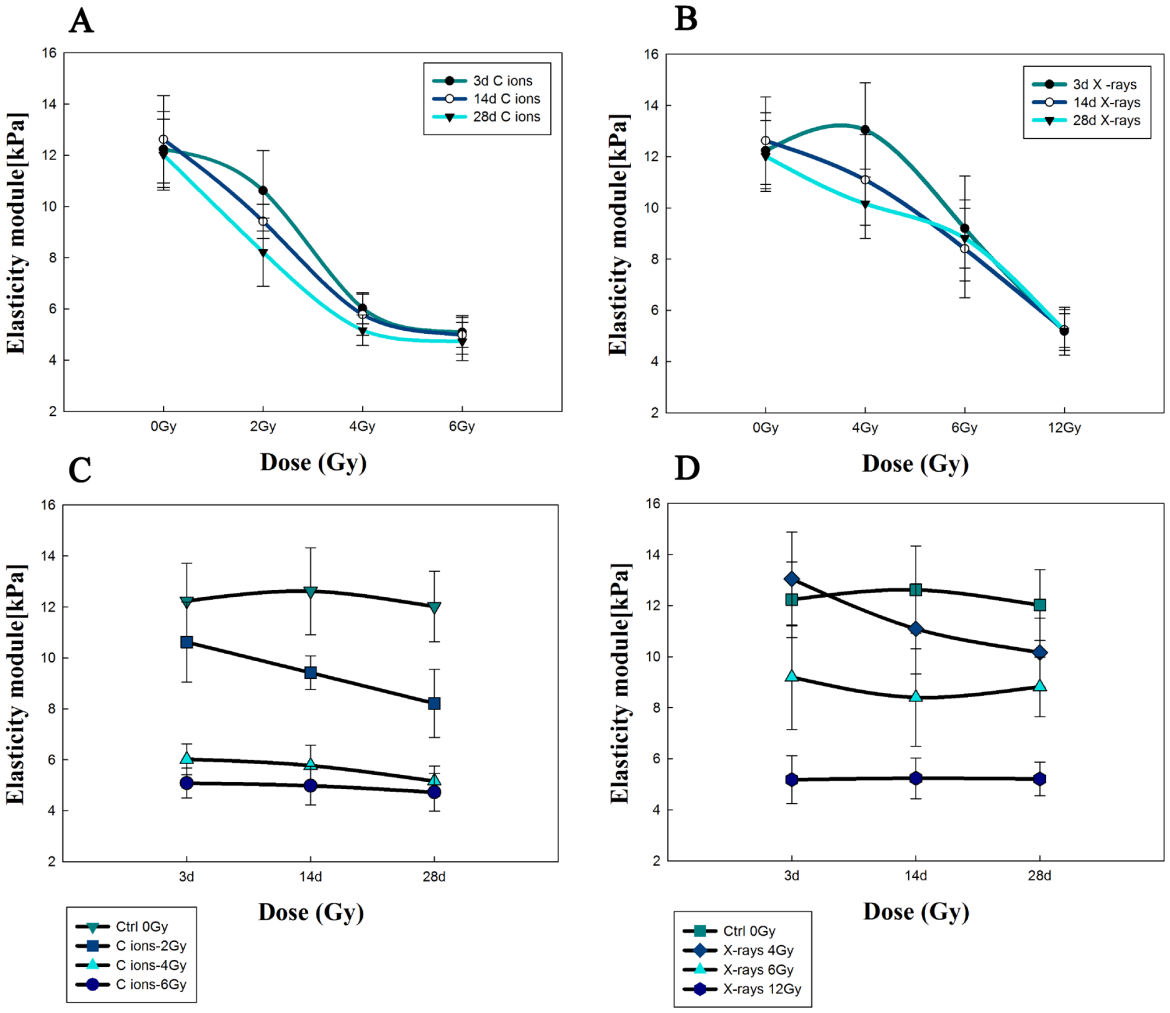
The change of elasticity modules of erythrocytes induced by C ions or X-rays radiation. And the groups displayed as a function of the dependent manner on dosage (**A** and **B**) and time (**C** and **D**). Error bars represent half-widths of the normal distributions fitted to the respective histograms.

The elastic modulus is often used to describe the mechanical properties of cells and other biological samples. The modified Hertz model fit well to the experimental data obtained by AFM measurement, and the calculated value of the elastic modulus was obtained using JPK Instruments data processing software. Stiffness measurements for the erythrocytes treated with different radiation doses were recorded relative to the reference hard material at 3, 14 and 28 days. The results are shown as histograms in **Figure 6** (including, carbon ion beams and X-rays), with the bin size reflecting the confidence interval of the measurements. The average values of the elastic modulus were obtained by fitting the Gaussian distribution to the histograms. Justification for using a normal distribution function for the analysis of the experimental data was provided by performing the Shapiro–Wilk normality test. This procedure indicated that all of the distributions from **Figure 6** were normal at the level of α = 0.05. Furthermore, the equality of variance was successfully verified using Levene’s test, and the statistical evaluation of the differences among the distributions was performed using analysis of variance (ANOVA). The ANOVA results showed that the means were different at the level of α = 0.01. The time dependence of the average elastic modulus is shown in **Figure 7**. The error bars in this figure represent half-widths of the normal distributions from **Figure 7**. It could be concluded that the elastic modulus of the phospholipid bilayer membrane in erythrocytes decreases slightly over time with exposure to large doses of radiation. Additionally, an interesting phenomenon is evident in the figure: with the increasing radiation doses, the elastic modulus also decreased. In the experiment, the changes in the elastic modulus were due to different radiation doses with CIR or X-rays, triggering injury to the membrane-cytoskeleton system.

**Figure 8:**
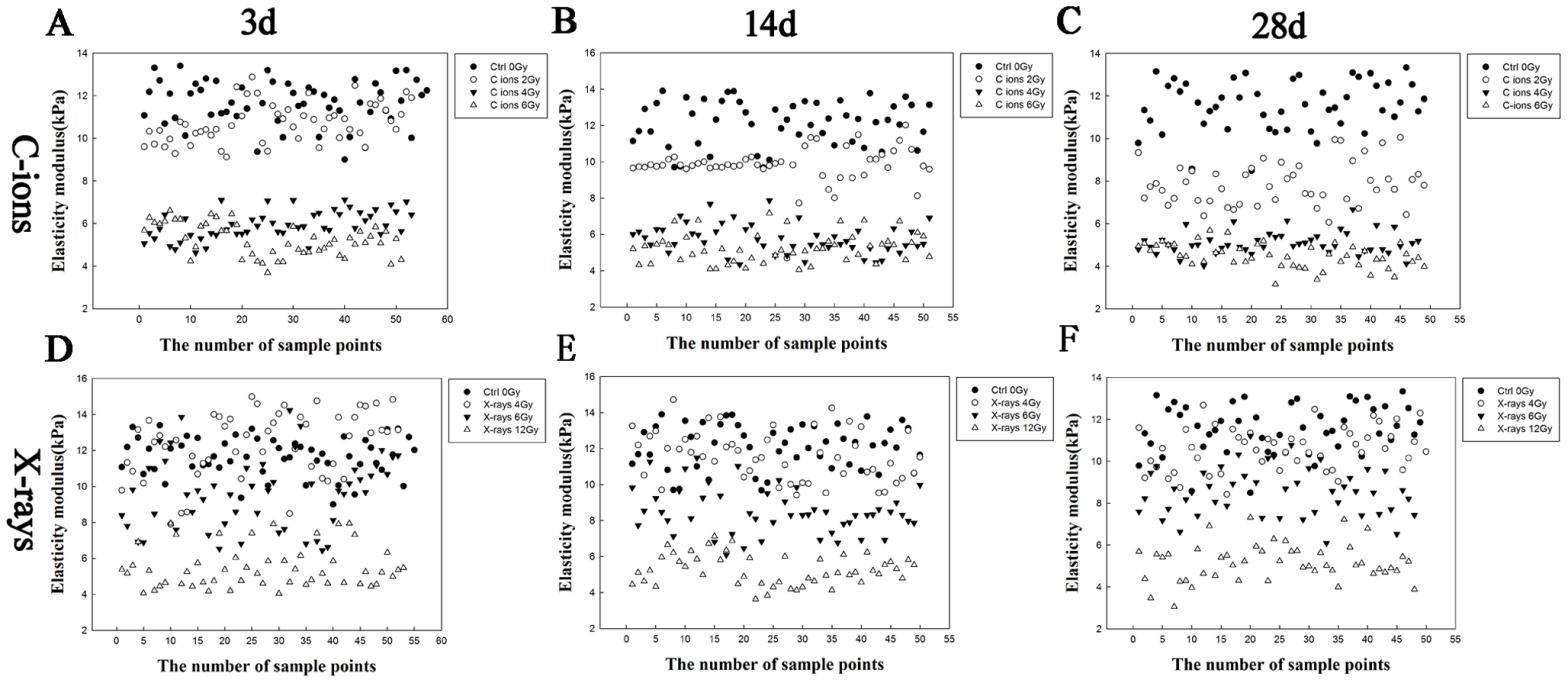
Variations of elasticity modulus for different doses of radiation in erythrocyte samples–control erythrocytes, and after suffering from the irradiation dose, the distribution effect of elasticity modulus was showed by scatters dots (**A–F**)**.**

**Figure 8** shows scatter plots of the elastic modulus values of the irradiated and non-irradiated groups over 3, 14, and 28 days calculated from the average of the approach force curves, and the results are shown as box-plots in which the scattered dots represent the effective sample point number. The data indicate that there were similar trends in variations of the elastic modulus according to the different doses of radiation in the erythrocyte samples, possibly indicating that the elastic modulus was proportional to the radiation dose.

### 3.4 Spectrin-α1 specifically participates in the morphological remodeling of irradiated erythrocytes

**Figure 9:**
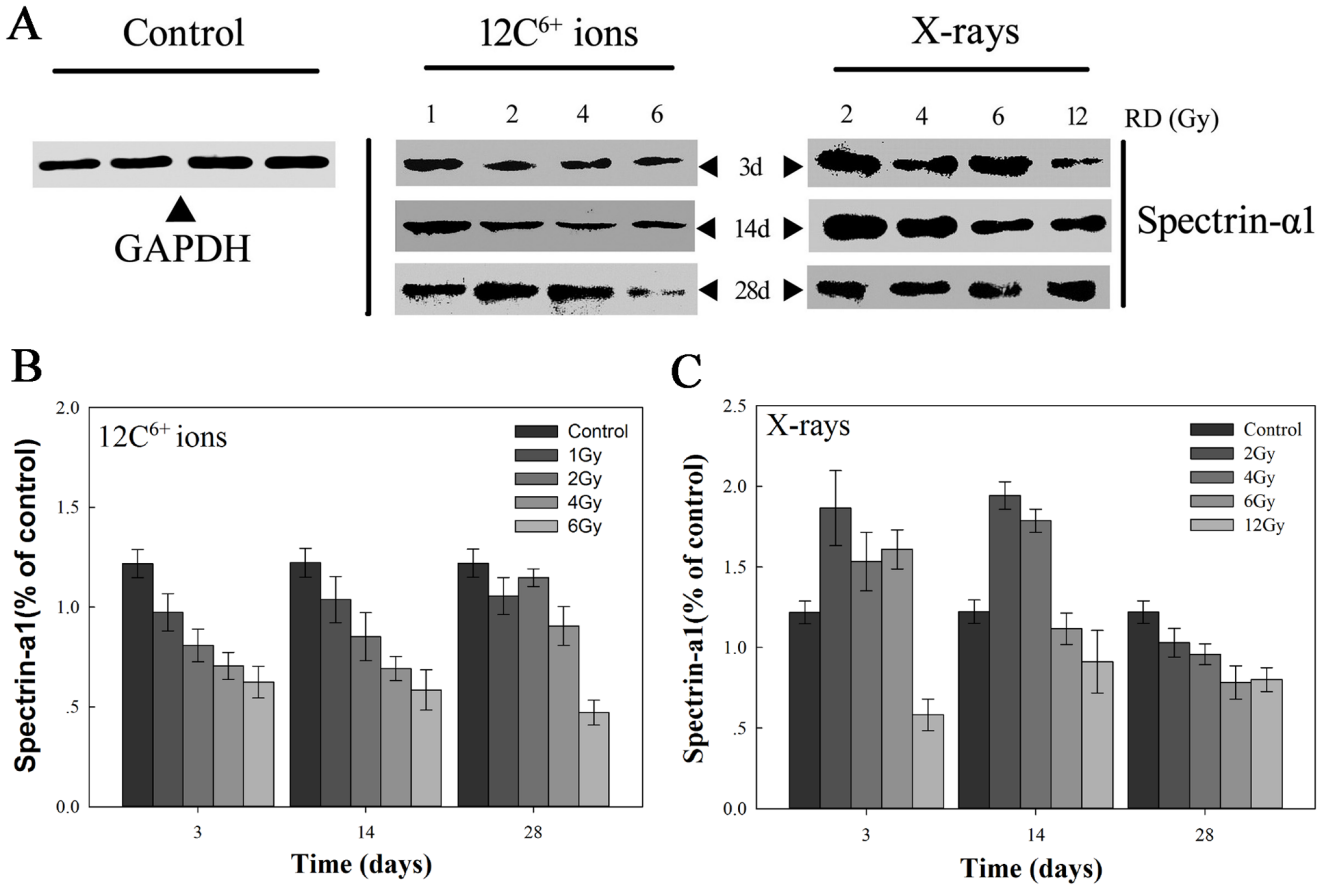
Spectrin-α1 is specifically targeted to drive erythrocyte skeleton impairment by C ions or X-rays radiation induction. Western blotting analyzed of the change of erythrocytes skeleton proteins spectrin-α1 expression after treated with radiation (**A**). (**B**) After C ions exposed at 3, 14 and 28d, the change of skeleton protein expression in different radiation groups. (**C**) After X-rays exposed at 3, 14 and 28d, and skeleton protein expression was also monitored by WB in different radiation groups. Error bars represent standard deviation from more triplicate valid data.

After exposure to CIR, the expression of spectrin-α1 protein significantly decreased in erythrocytes. With increasing radiation doses, spectrin-α1 protein expression was reduced. From 3 to 28 days after exposure to CIR, spectrin-α1 expression steadily decreased with increasing radiation doses. However, for X-rays, when small doses (<4 Gy) of radiation were utilized, spectrin-α1 expression was slightly augmented for a short time (approximately 14 days); with larger doses of radiation, spectrin-α1 expression appeared to be inhibited. As shown in **Figure 9**, the changes in erythrocyte morphology at the micro-nano scale level illustrate the integrity of the cell membrane and the stability of the cytoskeleton, which are dependent on protein conformation.

### 3.5 Comparison of the variation between the average of the elastic modulus and the mean density levels of spectrin-α1 caused by radiation

As indicated in **Table 2**, noticeable differences were observed for all of the data before and after irradiation. The mean density values of spectrin-α1 protein expression were significantly lower in the irradiated samples than in the non-irradiated samples (*p<0.05*), and the difference in the elastic modulus of both groups is clearly shown in **Table 2**. In particular, after exposure to CIR for 3 days, with increasing radiation dose from 2 Gy to 6 Gy, the mean density values of spectrin-α1 protein expression ranged from 0.99±0.16 to 0.75±0.15, which was significantly different from those of the control non-irradiated group *(p<0.05)*. However, at 14 days, the radiation-exposed cells showed mean density values of spectrin-α1 protein expression between 1.03±0.19 to and 0.71±0.17 with increasing radiation dose. At 28 days, the mean density values of spectrin-α1 protein expression ranged from 1.39±0.12 to 0.57±0.13. Furthermore, the mean elasticity modulus values of the irradiated group were reduced with increasing radiation dose from 10.62±1.57 kPa to 5.08±0.59 kPa after irradiation for 3 days; at 14 and 28 days, the average value of the Young’s modulus ranged from 9.42±0.66 kPa to 4.98±0.76 kPa and from 8.22±1.33 kPa to 4.72±0.74 kPa, respectively. For X-ray radiation, results similar to those obtained with CIR were observed (**Table 2**). Thus, in summary, the results indicate that when the micromorphology and microstructure of an erythrocyte change from a typically smooth, biconcave disc to a cell surface with corrugated, irregular, and rolling regional topography, changes in the elastic modulus and morphology occur in parallel with the irradiation process. Additionally, the fact is the elastic modulus values always increase when young cells become old cells in process, the form of change coexist in the process of erythrocyte changes by CIR induced, but the radiation induce the cell apoptosis that is a programmed cell death process along with the evolution of time, the activation of signaling molecules, then the series of cascading effects of transduction pathway [88], [89]. Specific mechanisms with age may be different from the mechanism of apoptosis, maybe there are some commonness, and also differences. Clearly, age-related changes have showed a transitions from physiological to pathological state, and the change of age relative to the apoptosis process is negligible. Therefore, the elastic modulus may function as a “biomarker” in the process and can be used to assess the harmful effects to erythrocytes or other cell types/tissues triggered by irradiation. The assessment of a single cell or group of cells may be sufficient to connect the biomechanical properties of cells and the protein molecular signaling cascade.

**Table 2:** Average values of Young’s modulus and mean density values of spectrin-α1 expression for the change of RBCs distributions according to different radiation groups.

Group	Spectrin-α1 protein expression(mean density)			Young’s modulus(kPa)		
	3d	14d	28d	3d	14d	28d
**Control**	1.21±0.07	1.22±0.62	1.22±0.64	12.23±1.48	12.62±1.71	12.02±1.38
**C ions 2Gy**	0.99±0.16^▾^	1.03±0.19^▾^	1.39±0.12^▾##^	10.62±1.57	9.42±0.66	8.22±1.33
**4Gy**	0.88±0.14^▾^	0.82±0.13^▾^	1.10±0.18^▾##^	6.02±0.60^Δ#^	5.77±0.80^Δ#^	5.17±0.60^Δ#^
**6Gy**	0.75±0.15^▾^	0.71±0.17^▾^	0.57±0.13^▾##^	5.08±0.59^Δ#^	4.98±0.76^Δ#^	4.72±0.74^Δ#^
**X-rays 4Gy**	1.86±0.26^▾^	2.17±0.14	1.16±0.14^##^	13.05±1.84	11.09±1.77	10.16±1.35
**6Gy**	1.95±0.20^▾^	1.36±0.17	0.95±0.17^▾##^	9.20±2.05^Δ^	8.40±1.91^Δ^	8.82±1.17^Δ^
**12Gy**	0.67±0.16^▾^	1.11±0.27^▾^	0.97±0.14^▾^	5.18±0.94^Δ#^	5.23±0.80^Δ#^	5.21±0.66^Δ#^

Errors are a standard deviation of the mean (SEM).

**Note,** ^Δ^ White triangles represents the statistical significance compared with the control group, ^#^the pound sign shows the plot as time goes on and the difference mean Young’s modulus(E) between each dose group (*mean ± SEM*). (for 3d, Control *vs* C ions-2Gy, p = 0.114, *p>0.05*; Control *vs* C ions-4Gy, p = 0.000, *p<0.01*; Control *vs* C ions-6Gy, p = 0.000, *p<0.01*; Control *vs* X rays-4Gy, p = 0.200, *p>0.05*; Control *vs* X rays-6Gy, p = 0.000, *p<0.01*; Control *vs* X rays-12Gy, p = 0.000, *p<0.01*; for 14d, Control *vs* C ions-2Gy, p = 0.000, *p<0.01*; Control *vs* C ions-4Gy, p = 0.000, *p<0.01*; Control *vs* C ions-6Gy, p = 0.000, *p<0.01*; Control *vs* X rays-4Gy, p = 0.243, *p>0.05*; Control *vs* X -rays-6Gy, p = 0.000, *p<0.01*; Control *vs* X rays-12Gy, p = 0.000, *p<0.01*;for 28d, Control *vs* C ions-2Gy, p = 0.095, *p>0.05*; Control *vs* C ions-4Gy, p = 0.000, *p<0.01*; Control *vs* C ions-6Gy, p = 0.000, *p<0.01*; Control *vs* X -rays-4Gy, p = 0.871, *p>0.05*; Control *vs* X rays-6Gy, p = 0.000, *p<0.01*; Control *vs* X rays-12Gy, p = 0.000, *p<0.01*). But ^▾^ blank triangles standards for the differences of spectrin-α1 protein average level (sp) and mean Young’s modulus (E) in different time points (*mean ± SEM*). The distinction was analyzed and we obtained an obvious statistical significance. ^##^Double pound sign shows the plot as time goes on and the difference of protein between each dose group. (for 3d, Control *vs* C ions-2Gy, p = 0.000, *p<0.01*; Control *vs* C ions-4Gy, p = 0.000, *p<0.01*; Control *vs* C ions-6Gy, p = 0.000, *p<0.01*; Control *vs* X rays-4Gy, p = 0.013, *p<0.05*; Control *vs* X rays-6Gy, p = 0.000, *p<0.01*; Control *vs* X rays-12Gy, p = 0.000, *p<0.01*; for 14d, Control *vs* C ions-2Gy, p = 0.000, *p<0.01*; Control *vs* C -ions-4Gy, p = 0.000, *p<0.01*; Control *vs* C ions-6Gy, p = 0.000, *p<0.01*; Control *vs* X rays-4Gy, p = 0.000, *p<0.01*; Control *vs* X rays-6Gy, p = 0.020, *p<0.05*; Control *vs* X rays-12Gy, p = 0.023, *p<0.05*; for 28d, Control *vs* C -ions-2Gy, p = 0.010, *p<0.05*; Control *vs* C ions-4Gy, p = 0.046, *p<0.05*; Control *vs* C ions-6Gy, p = 0.000, *p<0.01*; Control *vs* X rays-4Gy, p = 0.341, *p>0.05*; Control *vs* X rays-6Gy, p = 0.000, *p<0.01*; Control *vs* X rays-12Gy, p = 0.002, *p<0.01*).

### 3.6 Spatial correlation of the expression of cytoskeletal protein spectrin-α1 and the elastic modulus in irradiated erythrocytes

**Figure 10:**
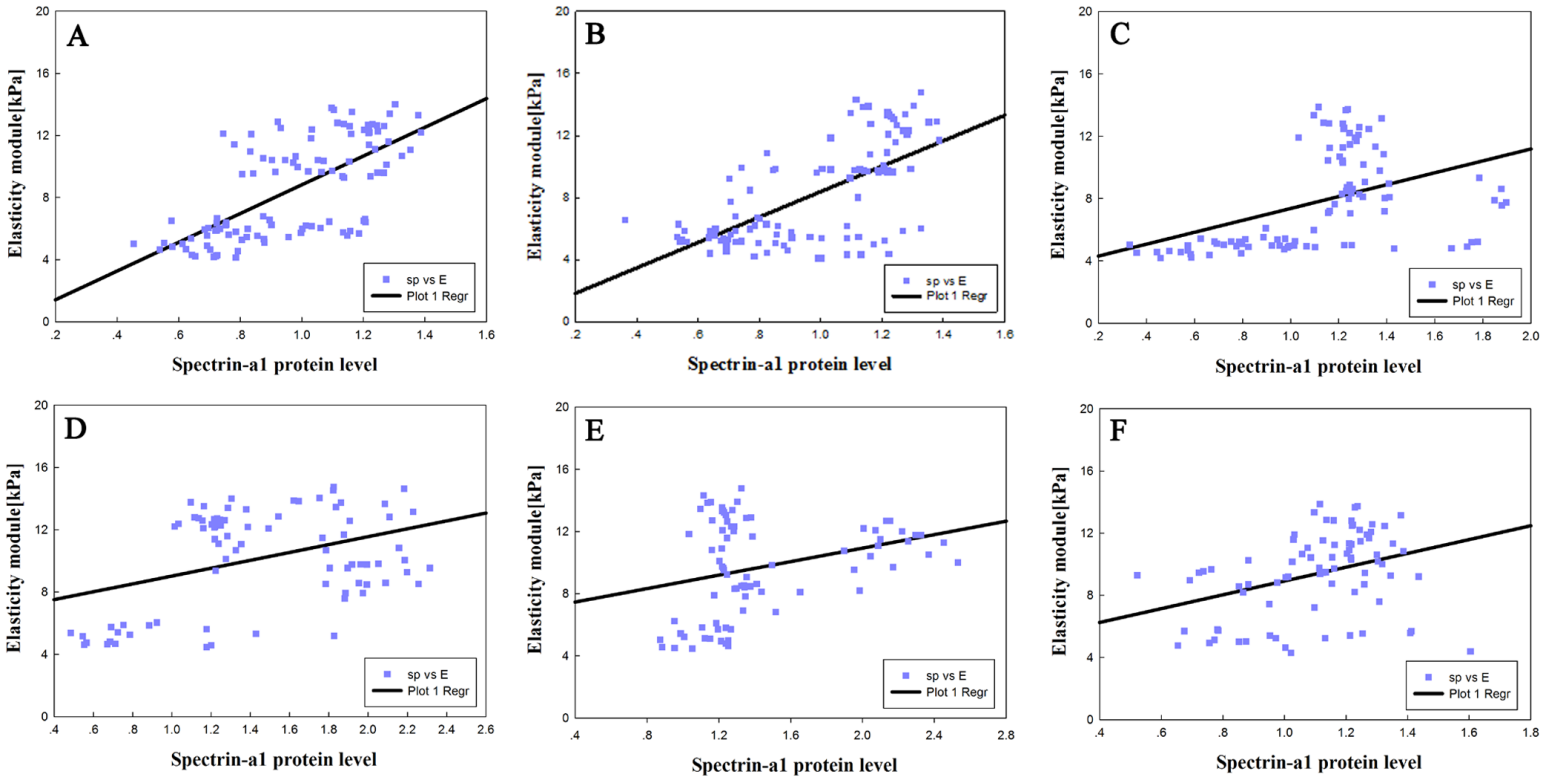
Spatial correlation of nanoscale modulus and spectrin-α1 protein expression with different doses of C ions or X-rays irradiated. With the increasing of radiation dosage, the relative protein expression of spectrin-α1 (sp) seemed more spatially correlated to elastic modulus (E) than low dose radiation (**A–F**). Comparison of correlation of the modulus in the same areas with Pearson’s testing. In control erythrocyte samples, there appeared to be non-correlation among the Young’s modulus in high-modulus regions, or low-modulus regions and the expression of spectrin-α1 protein. After treated with the radiation, the average elastic modulus was reduced and areas of cell membrane appeared to be more correlated with the modulus. Through the Pearson’s correlation coefficient between modulus and spectrin-α1protein level by western blotting detection. Reflective of these findings, after exposure by carbon ion beams at 3 days, Pearson’s correlation coefficient (r) between modulus and spectrin-α1 protein expression were relatively strong but significant (r_3c_ = 0.678, *p<0.01*) compared with others time points; At 14 days irradiated by carbon ions, Pearson’s correlation coefficient between them showed positive correlation (r_14c_ = 0.639, *p<0.01*); at 28 days irradiated by carbon ions, the correlation coefficient was (r_28c_ = 0.438, *p<0.01*). For X-rays, there were similar radiation effects with carbon ion beams in different groups, respectively, at 3, 14 and 28 days, the correlation coefficient were (r_3x_ = 0.390, *p<0.05*), (r_14x_ = 0.301, *p<0.01*) and (r_28x_ = 0.353, *p<0.01*).

After the experimental groups were exposed to CIR or X-rays, the spatial relationships between spectrin-α1 protein expression and the elastic modulus in erythrocytes were examined. We correlated the value measured for the elastic modulus of the erythrocyte membrane using BT-AFM with the expression of the cytoskeletal protein spectrin-α1. Qualitatively, the elastic modulus appeared to be positively correlated with the relative expression of spectrin-α1 (i.e., a high modulus was observed in regions with higher protein expression in the context of irradiation) and was also correlated with radiation dose (low modulus in regions with lower protein level in the context of irradiation). Additionally, there was a special dose-response relationship between the elastic modulus and the radiation dose: when the irradiation dose increased, the elastic modulus increased as well (**Figure 10**). Based on these findings, Pearson’s correlation coefficient (r) was calculated between the elastic modulus and the relative level of cytoskeletal protein spectrin-α1. In control erythrocyte samples, there appeared to be no correlation among high-modulus regions, low-modulus regions and spectrin-α1 protein expression. After treatment with radiation, the average modulus was reduced, and the area of the cell membrane appeared to be more correlated with the modulus. Reflecting these findings, after exposure to CIR for 3 days, Pearson’s correlation coefficient (r) between the modulus and spectrin-α1 protein expression was relatively strong and significant (r_3c_ = 0.678, *p<0.01*) compared with the other time points. At 14 days of irradiation with CIR, Pearson’s correlation coefficient showed a positive correlation (r_14c_ = 0.639, *p<0.01*); at 28 days of irradiation by CIR, the correlation coefficient was also positive (r_28c_ = 0.438, *p<0.01*). For X-rays, there were radiation effects similar to those of CIR among the different groups: at 3, 14 and 28 days, the correlation coefficients were (r_3x_ = 0.390, *p<0.05*), (r_14x_ = 0.301, *p<0.01*) and (r_28x_ = 0.353, *p<0.01*), respectively.

## Discussion

### The inherent mechanical characteristics of cells are an important manifestation of their physical state under external perturbation

The mechanical properties of individual cells have been regarded as unique indicators that can constantly reflect the changes in their states caused by cellular events and pathological conditions. Simply put, alterations in biological activity or transformation of cell states can also trigger changes in the mechanical properties of cells. In particular, alterations in cell stiffness or the elastic modulus have been used as biological markers for cellular phenotypic events and diseases [90]. The mechanical properties of cells are largely attributable to the cytoskeleton components and the cytoskeletal architecture, which maintain the basic form of cells. Thus, for the abovementioned changes in a cell’s life cycle, visualizing the changes in the mechanical properties of cells exposed to some type of stimulus and the association of these changes with the biomechanics of cellular events have very important significance. Previously, the spectrin skeleton of erythrocytes was studied as a model tethered membrane [91], and many results have suggested that spectrin is a vital scaffold protein involved in signaling pathway organization, influencing changes in cytoskeletal structure and controlling membrane function.

### Radiation induces changes in erythrocyte stiffness during morphological remodeling

In the present study, the mechanical properties of erythrocytes were assessed by combining the use of biological-type AFM, cytological analysis of samples of peripheral blood and optical microscopy of bone marrow after irradiation by CIR or X-rays. The experimental results suggested that there was a clear relationship between changes in cell morphology and variations in mechanical properties; this relationship was mainly manifested in the following respects. First, in terms of morphology, a normal erythrocyte is a biconcave disc resembling a round cake, has a uniform size, has no nucleus, appears as a bilaterally indented sphere of approximately 4–8 µm in diameter (in mammals; in humans, 6–9 µm), has a thickness of approximately 1.0–2.5 µm, and has a relatively uniform, flat, and smooth membrane surface that is structured and maintains some continuity. However, after 3 days of irradiation with CIR, these cells underwent shrinkage and became irregular, and some exhibited abnormal spines or ruffles on the edges; additionally, the membrane area exhibited discontinuities and an undulating surface (**Figure 1**). Furthermore, the cytological smear method was used and analyzed with the NIH Image J software to reveal changes in the cell form in peripheral blood samples and in the proportions of different cell types in the marrow. The inherent parameters, such as the length, width, perimeter, thickness, ROI area and volume were quantitatively analyzed. Measurement of the results showed that the average value of the length was reduced from approximately 7.25±0.13 µm to 5.96±0.24 µm, the average value of the width was reduced from approximately 6.12±0.10 µm to 5.40±0.10 µm, the average value of the perimeter was reduced from approximately 584.14±36.78 µm to 519.0±45.65 µm, the ROI area was reduced from approximately 20517.44±3651.02 µm^2^ to 17317.20±2419.97 µm^2^, and the average volume was reduced from approximately 41.03±7.30 µm^3^ to 34.63±4.84 µm^3^. Additionally, the average thickness of erythrocytes in mammals is in the range of approximately 1.0–2.5 µm. Using an optical microscope, an average thickness value of 2.0 µm was found and used to calculate the relative volume of the erythrocytes. Additionally, using the tangible cell counting method to analyze the proportion of erythroid cells in BM, there was a mild myeloproliferative effect of small doses of radiation. Furthermore, given the priority of the early development of erythroid cells, the pronormoblasts and basophilic erythroblasts seemed to demonstrate a tendency to increase with increasing absorbed CIR doses; however, this change was not obvious in the X-ray radiation group. Taken together, these results indicated that when the radiation dose increased, there was an obvious inhibitory effect on BM; thus, the proportions of polychromatophilic erythroblasts and normoblasts decreased significantly with both types of irradiation (**Figure 2** and **Figure 3**)**.**

Most importantly, BT-AFM was employed in this study as a powerful and versatile tool to study the mechanical properties at the micro-nano scale for the analysis of fine surface structure changes on erythrocytes, emphasizing the role of the cell membrane and the integrity of the membrane in cell mechanical stability following radiation exposure. This study investigated the microstructural and mechanical properties of erythrocytes and quantified the deformation and damage to the membrane skeleton. In particular, as shown in **Figure 5**, AFM topographic images reveal the fine variations induced by radiation, and the images were obtained of shrunken, irregular, ruffled-edge shapes and progressively discontinuous membrane structures. Next, a conclusion can be drawn based on the relative elastic modulus or stiffness, which depends on the precise measurement of the mechanical properties of the cell membrane. The mean elastic modulus of erythrocytes irradiated with increasing CIR doses was decreased to between 10.62±1.57 (kPa) and 5.08±0.59 (kPa) at 3 days after irradiation; at 14 and 28 days, the average values of the elastic modulus ranged from 9.42±0.66 (kPa) to 4.98±0.76 (kPa) and 8.22±1.33 (kPa) to 4.72±0.74 (kPa), respectively. For X-ray radiation, similar results were observed to those obtained with CIR. Thus, fundamental alterations in erythrocyte stiffness were triggered by the different radiation types, at different times and with different doses. Furthermore, the combination, the distribution, and the changes in stiffness were also different, as shown in **Figures 6** **,** **7** and **8**. This strategy allowed us to perform nanobiological analysis of the erythrocytes after irradiation, further improving our knowledge of the aspects and dynamics of the mechanical properties of erythrocytes.

### Spectrin-α1, a neofunctionalized cytoskeletal protein of erythrocytes, is involved in mediating changes in erythrocyte stiffness during the morphological remodeling process induced by radiation

**Figure 11:**
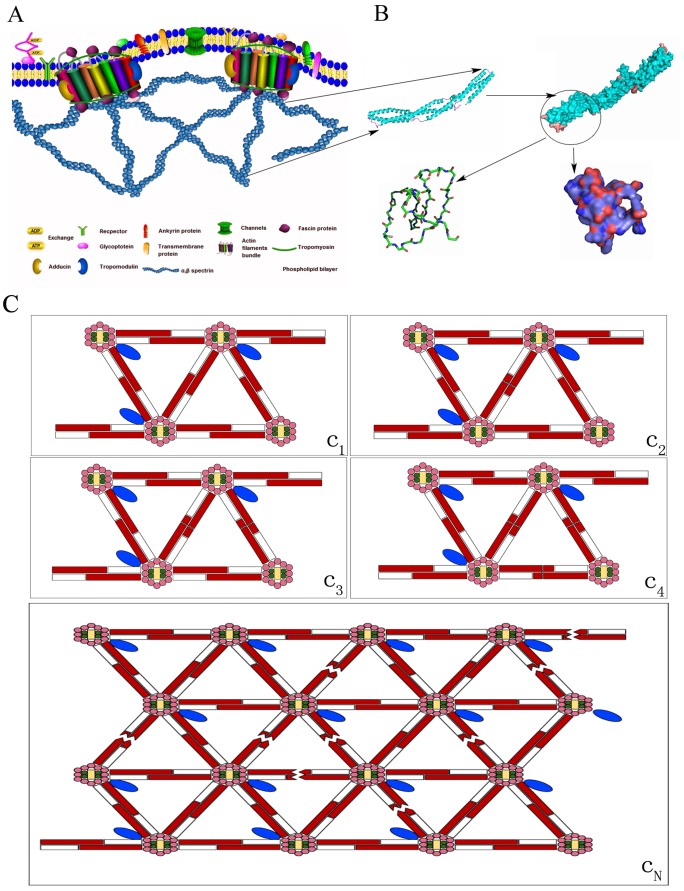
This picture shown the assembly structure of the scaffold protein spectrin and its spatial configuration, and were proposed a deformation hypothesis of our model. (**A**) The figure was a schematic diagram of spectrin and other cytoskeletal molecules. (**B**) Stereoview ribbon diagram of the overall structure of spectrin protein in all the repeats around the kink region. The first and second repeat (α, β subunit) were shown in light blue, and the linker region between repeats in purple, Residues were numbered according to the SWISS-PROT NP_003117.2 entry, and the residues from 1 to 2149 aa of Homo sapiens. Lastly, the software of the PyMOL Molecular Graphics Systerm was used to simulate the molecular structure, including a complete of the molecular structure and partial structure were displayed. (**C**) A series of random deformation process will be shown. The figure was α-spectrin in dark red band, and β-spectrin with a white band, the shape of saw tooth was rupture boundary. C_1_ represented for completely without any deformation, then, C_2_, C_3_ and C_4_ respectively shown that from a set of skeleton to three groups of skeleton were broken. But the case of C_N_ manifested randomly the fracture of cytoskeletal protein. Thus, this phenomenon could reflect the uninterrupted and dynamic of changes from small deformation to large deformation during the radiation, and more details were explained in the article.

The membrane skeleton, a dense proteinaceous network, is thought to be responsible for the remarkable morphological changes and mechanical properties of erythrocytes [92], allowing them to withstand and respond to different mechanical stresses and tension caused by external perturbations experienced throughout their 120-day lifespan [93]. The basic shape of an erythrocyte can be visualized by observing the spectrin-containing sub-membranous cytoskeleton because this structure preserves the outline of the cell. The spectrin molecules form pentagonal or hexagonal arrangements, which are composed of tetramers of spectrin subunits associating with short actin filaments at either end of the tetramer. These short actin filaments act as junctional complexes, allowing the formation of a hexagonal mesh and scaffolding. This assembly configuration not only plays an important role in the maintenance of plasma membrane shape, integrity, and cytoskeletal structure, influence influencing deformability or resulting in damage [90], [94], [95] and finally determining biological behavior and functional aspects [96]–[99]; it can also produce a rapid response and break the tetramers [100]. A dysfunctional underlying membrane skeleton results in the erythrocyte membrane being partially devoid of a spectrin-actin network, no longer existing as a ghost, and causing disruption of the unique arrangement of spectrin, F-actin, protein 4.1 and ankyrin, with direct and indirect connections to the membrane bilayer with its immersed integral proteins [101] (**Figure 11**). Consequently, the distribution of membrane tension is regulated by signaling molecules through the activated complex signaling transduction pathway to mediate the biochemical reaction between target proteins or molecules [102]–[104] and triggering exquisite changes in cell shape or membrane damage [105]. Similarly, it was reported that the skeleton began to fragment and became a small, approximately 50-nm inverted vesicle [52], producing changes in cell shape and stability regarding the filamentous network. Consequently, the importance of this structure is highlighted by the above discussion noting that spectrin is a structural platform for the stabilization and activation of membrane channels, receptors and transporters in mammals; however, it is also closely related to cell physiology and pathology [106], [107].

Many studies have shown that the measurement of mechanical properties in biological samples can explain the biomechanical mechanism of organism units in various physiological and pathological states. Recent evidence suggests that shear can induce the unfolding of spectrin within erythrocytes [108], forcing changes in protein structure by the unfolding of specific regions, and that the spectrin repeat unit can be subjected to reversible unfolding and refolding using 20-pN forces [109]. Regarding the erythrocyte cytoskeleton, spectrin comprises a tetrameric or higher oligomeric protein arranged as antiparallel filamentous heterodimers of α and β subunits [52], [110], which is easy to open or fold; moreover, spectrin is organized in a scaffold located at the intracellular side of the plasma membrane in eukaryotic cells, and is linked to several integral membrane proteins through a helical repeating unit. Additionally, spectrin and a few other proteins assemble into a building block and share a common structural fold with all of the spectrin repeat units [52]. Ultimately, the crystal structure of the assembled protein shows that it maintains an uninterrupted helical structure [111], creating a steady state for the cytoskeleton meshwork and maintaining the native cell shape. In the present study, the expression of the cytoskeletal protein spectrin-α1 was analyzed using a quantitative immunoblot technique after irradiation, and the results showed that CIR or X-rays can cause the expression of spectrin-α1 to decrease significantly in irradiated erythrocytes. This effect relied on different radiation doses, and the data obtained showed a relationship between the expression of cytoskeletal protein spectrin-α1 and changes in cell stiffness in the irradiated group, as well as a negative correlation regarding changes in erythrocyte stiffness during the morphological remodeling process. Additionally, regarding the AFM experimental data, examination of these changes in mechanical properties by the response to force is one of the most straightforward visual indices to evaluate the membrane damage mechanism caused by radiation, assess the overall performance of protein conformation and the protein grid in the erythrocyte membrane skeleton, and increase the understanding of the radiation-induced morphological remodeling-cytoskeletal remodeling process.

### Erythrocyte stiffness during morphological remodeling can be used as a biomarker to estimate the adverse effects induced by ionizing radiation

Based on the established parameters, the present study evaluated the roles of cell morphology and cell stiffness during the erythrocyte morphological remodeling process to determine the adverse effects on erythrocytes following exposure to radiation. The influence of the combination of cell signaling with the interactions of membrane phospholipids in vertical force interactions, horizontal interactions, and lateral network interactions of the spectrin structure were emphasized in the current study. Thus, it is necessary to clarify two basic problems of the mechanics induced by the effects of radiation. The first problem is the relationship between the stability of the membrane-associated cytoskeleton and changes in the mechanical properties (e.g., the stiffness or elastic modulus and the viscosity) of the erythrocytes. Another problem is that the shear stress or tension created by the effects of deformation or damage after radiation might influence how the radiation alters the cell behavior in various mechano-signaling processes, such as changes in cell shape, cell migration, cell movement and cell apoptosis. Thus, to solve these two problems, a link must be created between the biochemical molecules (proteins) and the response to force (cell stiffness) to improve our understanding of why mechanically induced changes in the protein structure within erythrocytes are likely to be important not only to cell deformability or damage but also to various mechano-signaling processes [35], [112].

When the erythrocytes were subjected to radiation, the cells first responded to the radiation at the cellular level. Next, the effect was further expanded via a signaling cascade starting at the level of proteins and molecules. Similarly to a scaffold protein, spectrin functions as a supporting protein of the erythrocyte membrane skeleton. Spectrin can organize and mediate signaling molecules into particular domains of specific signaling pathways that depend not only on the targeting affinity of proteins of the signaling pathway but also on the circumstances under which the expression of these signaling pathway components were increased and position these components in response to the radiation that activated the signal transduction pathway. Next, using this procedure, membrane proteins (comprising membrane channels, receptors and adhesion molecules) on the erythrocyte surface were activated to recruit target proteins to a specified location, organize them by affinity, and bind them into large biological macromolecular complexes. However, for spectrin proteins, posttranslational protein modifications of phosphorylation and dephosphorylation are involved in positioning and recruiting target proteins [52], resulting in a disturbance of the dynamic balance of the formation and depolymerization of scaffolding proteins [113]. This disturbance leads to the openings in the structure, rotation, and fracture, causing cytoskeleton rearrangement and collapse of the membrane skeleton due to radiation effects, in addition to directly affecting and modulating the mechanical stability of the erythrocyte plasma membrane. The direct effects manifest as uneven changes in the regional distribution of tension forces on the cell membrane surface; thus, the deformation or damage to the erythrocyte morphological remodeling process can be assessed.

However, for morphology-cytoskeleton remodeling, tension or stress forces are always involved in this complex coupling process. The cascade effects in the pathway are caused by biological composite macromolecules that can produce changes in the cytoskeleton-associated proteins in the spatial conformation of the erythrocyte–e.g., spectrin. There is a rigorous procedure to facilitate the interaction and fine-tune the activity and crosstalk among the proteins within the entire assembly. This targeted protein expression shows increased biological activity, which influences the activity of downstream proteins under their control, until the dynamic balance of the molecules is again coordinated. Thus, different molecular assemblies in different regions operate on the functions or implement local “microdomains” of the cell. However, the complexity of this response relies on several components of the membrane skeleton, and the stiffness of spectrin upon phosphorylation may permit the formation of many junctions to allow fast, reversible rearrangement of the spectrin network upon large deformations, thus indirectly modulating the mechanical stability of the membrane [114]. Certainly, the membrane stability depends largely on resultant forces in three directions to transfer the spatial effects of deformation or damage to the scaffolding proteins. If the protein chains are to be studied as a geometric unit, some of the mechanical behavior must be evaluated under the stress-induced load bearing of the protein structural unit (**Figure 11**). Additionally, the surface density of the filaments decreases from the center to the edge of the membrane-skeleton, and the overall thickness of the membrane-skeleton also increases from the edge of the cells (54 nm) to the center (110 nm) [115], suggesting a distribution imbalance between the surface density and the membrane-skeleton thickness, leading to tension of the membrane surface. The stress of the cytoskeleton protein chain as a unit inside the cell is unevenly distributed in partial regions or across the entire cell span. The direction of force is along the three axes; thus, the spectrin tetramers can dissociate transiently into dimers, and some of the repeating units of the spectrin chains can unfold. The dissociation of the spectrin tetramers leads to relatively unimpeded translational diffusion of transmembrane proteins and may be expected to allow them to form irregular clusters [116]. Therefore, in horizontal-plane interactions, including spectrin dimers, tetramers, hexamers and octamers, and even higher oligomeric proteins, the tension generates the interactions between the protein chains. In vertical-force interactions, the direction of the force mainly originates from two complexes, which produce shear force by the junctional complex and the AE1-ankyrin complex. The force of the lateral network interactions is more complex and presents numerous binding sites and binding proteins for a broad diversity of partners. There are mainly F-actin binding sites, actin-binding sites, and tropomyosin-, tropomodulin-, adducin-, and dematin-binding proteins participating in local regions of the network to dynamically change the mechanical properties, thus facilitating reversible deformations that the erythrocyte undergoes in different external environments [117], [118] (as shown in **Figure 11**). The topographic and mechanical mapping of single components at the cytoplasmic face reveal that, surprisingly, the erythrocyte membrane mechanics are regulated by spectrin phosphorylation related to the metabolic state and the assembly of structural elements in the membrane [111]. Of course, to a certain extent, the local distortion or ratio of damage in cells is mainly determined by the radiation dose. Thus, the spectrins can fully dominate the deformation or damage of the cytoskeletal meshwork in the morphological remodeling process [119]. Considering the current knowledge that spectrin proteins can trigger a biological effect sufficient to change the local or overall cell morphology, as well as the mechanical and structural properties that are changed, few data are available on the interactions of spectrin with several participants in a particular pathway, organizing it into a chain of signaling events. However, according to the present study, the spectrins could be considered docking proteins in the morphological remodeling process induced by radiation.

## Conclusion

In summary, our results suggest that CIR could influence the amount of components and reorganize the spectrin-α1 protein of the membrane-skeleton system, as well as affect the distribution of the regional tension or stress (force), resulting in changes in the surface topographical microstructure and mechanical properties (cell stiffness) in the erythrocyte morphological remodeling process. Additionally, we show how spectrin-α1 protein could be used as a biomarker to detect erythrocyte membrane deformation or damage and how this protein promotes the study of the biomechanical properties of signaling domains extending from the cell surface to deeper parts within the erythrocytes. These findings may bridge the gap between numerous interdependent behaviors of biological molecules and their mechanical properties to reveal the effects of deformation or damage or to assess the adverse effects on erythrocytes induced by radiation, thereby providing an inherent force-biochemical coupling analytical approach. The use of heavy ion radiation (^12^C^6+^ ions) has been proven to possess unique biological advantages in radiation therapy. Incidentally, we also suggest using heavy ion beam radiation pretreatment for the storage of erythrocytes, an application that has not yet been reported in the literature.
